# Endotracheal intubation with a video-assisted semi-rigid fiberoptic stylet by prehospital providers

**DOI:** 10.1186/s12245-014-0045-0

**Published:** 2014-11-26

**Authors:** Derek R Cooney, Charles Beaudette, Brian M Clemency, Christopher Tanski, Susan Wojcik

**Affiliations:** Department of Emergency Medicine, SUNY Upstate Medical University, 550 East Genesee/EMSTAT Center, Syracuse, NY 13202 USA; Department of Emergency Medicine, School of Medicine and Biomedical Sciences, SUNY University at Buffalo, 462 Grider Street, Buffalo, NY 14075 USA

**Keywords:** Airway, Clarus Video System, Emergency, EMS, EMT, Fiber optic, Fiberoptic, Laryngoscopy, Levitan, Optical, Prehospital, Shikani, Stylet, Video

## Abstract

**Background:**

Emergency medical technicians intubate patients in unfamiliar surroundings and with less than ideal positioning. This study was designed to evaluate advanced life support (ALS) emergency medical technicians' (EMTs) ability to successfully intubate a simulated airway using a video-assisted semi-rigid fiberoptic stylet, the Clarus Video System (CVS).

**Methods:**

ALS EMTs were first shown a brief slideshow and three example videos and then given 20 min to practice intubating a mannequin using both the CVS and standard direct laryngoscopy (DL). The mannequin was then placed on the floor to simulate field intubation at the scene. Each participant was given up to three timed attempts with each technique. Endotracheal tube position was confirmed with visualization by one of the study authors. Comparisons and statistical analysis were conducted using SPSS® Statistics 21 (IBM®). Demographics and survey results were also collected and analyzed.

**Results:**

The median total time for intubation was 15.00 s for DL and 15.50 s for CVS revealing no significant difference between the two techniques (*p* = 0.425), and there was no significant difference in the number of attempts required to successfully place the endotracheal tube (ETT) (*p* = 0.997). Demographic factors including handedness and eye dominance did not seem to affect outcomes. Participants reported a relatively high level of satisfaction with the CVS.

**Conclusions:**

ALS EMTs were able to obtain intubation results similar to those of their usual direct laryngoscopy technique when utilizing a video-assisted semi-rigid fiberoptic stylet with very limited instruction and experience with the device. The CVS technique warrants further study for use as an alternative to DL and video laryngoscopy in the prehospital difficult airway scenario.

## Background

Advanced life support (ALS) emergency medical technicians (EMTs) provide advanced airway management in the prehospital environment on a regular basis in most emergency medical services (EMS) systems in the United States and in many locations around the world where physicians are not the primary care provider. Prehospital intubation has been found to be somewhat challenging, and some authors have reported missed (esophageal) intubation rates ranging from 5.8% to 17% [1-4]. One meta-analysis of 57,132 patient encounters showed an intubation success rate of 75.9% for non-physician prehospital intubators [5]. Difficult anatomy has been reported as the primary cause of failed intubation by prehospital providers in 20% of the cases, along with clenching (49%) and airway obstruction (10%) [6]. Paramedics may be intubating on the floor in nearly 60% of the cases, and a cadaver study showed that the first pass success in this position was 77.4% compared to 86.9% when at an elevated stretch position [7].

**Figure 1:**
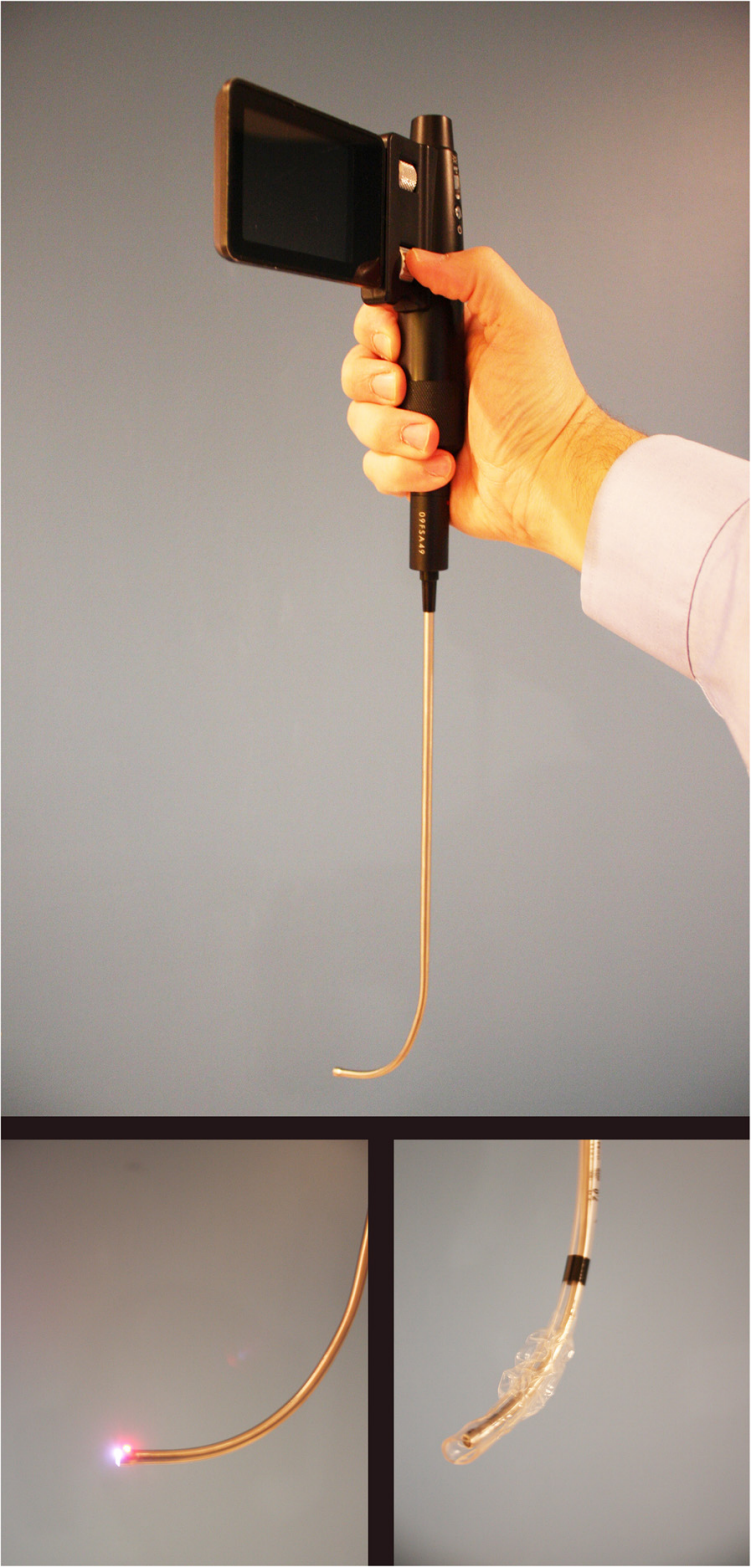
**Endotracheal tube loaded on the Clarus Video System.**

The Clarus Video System (CVS) is a video-assisted semi-rigid fiberoptic stylet that displays a view of the airway on a video screen attached to the side of the CVS and has been shown to be a useful device for difficult airway management [8-10]. The endotracheal tube is loaded on the short semi-rigid malleable metal stylet with fiberoptic distal light source just inside the tip of the tube (Figure 1). This study was designed to evaluate the ability of ALS EMTs to successfully intubate a normal airway in a simulated field environment with the CVS after providing only a brief introductory tutorial on the operation of the device.

### Hypotheses

1) Participants will achieve successful intubation in the same number of attempts when utilizing the CVS. For the purpose of this study, a difference of one in number of attempts would be considered a minimal clinically important difference (MCID). 2) The sum of the time for intubation attempts will be the same when using the CVS. For the purpose of this study, a difference in the total time of greater than or equal to 10 s would be considered the MCID. 3) ALS EMTs will be satisfied with the CVS and perceive it to be useful to their practice.

## Methods

A sample of volunteer ALS EMTs, certified and licensed by New York State (United States) at the ALS level, was first shown a brief video presentation on the use of the CVS. For a MCID of one attempt, 90% power and alpha = 0.05, the sample size required was 28. For a MCID of 10 s in the total time, 90% power and alpha = 0.05, the sample size required was 65.

**Figure 2:**
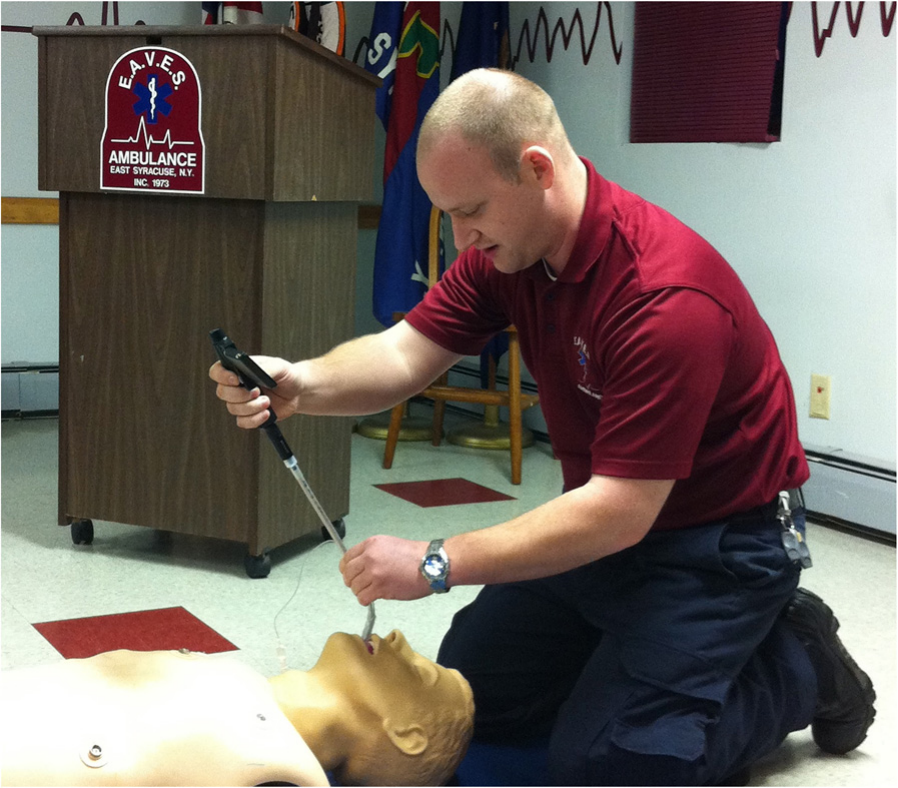
**ALS provider intubating the mannequin positioned on the floor.**

The video was 8 min and 22 s long and was followed by a hands-on period of familiarization lasting approximately 10 min during which participants were allowed to practice intubating the mannequin (Laerdal® Airway Management Trainer, Wappingers Falls, NY, USA) in a group with a standard 7.5-mm endotracheal tube (Rusch: Teleflex Medical, Research Triangle Park, NC, USA), using both the CVS and standard direct laryngoscopy (DL). During both the familiarization and testing phases, the mannequin was situated on the floor to simulate an out-of-hospital intubation scenario. Instruction on utilizing the CVS alone (without the use of a laryngoscope) was given describing a midline approach with the device. Alternative approaches to the use of the CVS were not utilized. Random selection (coin flip) was used to determine which technique would be used first by each participant. Each participant was given up to three attempts to intubate with each technique with the mannequin positioned on the floor (Figure 2). The attempts were timed from picking up the device, until the stylet (either the CVS or a standard malleable stylet) was removed from the endotracheal tube (ETT) in the mannequin. Location of the ETT after each attempt was confirmed utilizing the CVS to visualize the larynx after both techniques. When the participant was successful with either technique, they then utilized the other technique. If unsuccessful after three attempts with either device, the participant was instructed to move to the other technique. After completion of the intubation attempts, participants were asked to complete a demographics and survey form which included self-reported age, sex, eye dominance, handedness, and type of primary agency they work for. They rated their satisfaction with the CVS, perceived usefulness for their practice, and the effectiveness of the tutorial with 0 = not at all and 10 = completely.

Data was entered into SPSS® Statistics 21 (IBM®) and analyzed to determine the results for each technique. Median and percentile times for the first attempts were determined and compared with the Mann-Whitney test. The total combined time of attempts required for successful intubation was determined and analyzed with the Wilcoxon signed-rank test. The number of attempts was analyzed utilizing the chi-squared test. Potential impact of demographic factors was analyzed using Mann-Whitney and Kruskal-Wallis tests.

## Results

The sample size was 81 ALS EMTs which exceeded the needed sample sizes to achieve 90% power for hypothesis 1 (sample needed = 28) and hypothesis 2 (sample needed = 65). ALS EMTs were able to successfully place the ETT on the first attempt 95.1% of the time with DL and 96.3% of the time with the CVS. Two participants required two attempts and two required three attempts with DL. Two participants required two attempts with the CVS and one participant used all three attempts and was unable to intubate with the CVS. There was no significant difference in the number of attempts required to successfully place the ETT (*p* = 0.997) (Table 1). The median times for the total time of attempts for intubation were 15.00 s (range: 4.00 to 104.50 s) for DL and 14.50 s (range: 6.10 to 61.00 s) for CVS revealing no significant difference between the two techniques (*p* = 0.104) (Table 2). The median time for the first attempt was the same as the total time, and the minimums, maximums, and percentiles are listed in Table 3. Demographic information is reported in Table 4.

**Table 1:** **Attempts required to successfully intubate (** ***n*** = **81 participants)**

	**One attempt**	**Two attempts**	**Three attempts**	**Failed to intubate**
DL	95.1% (77)	2.5% (2)	2.5% (2)^a^	0% (0)
CVS	96.3% (78)	2.5% (2)	1.2% (1)^b^	1.2% (1)

^a^Both participants successfully intubated with DL on the third attempt; ^b^One participant who required three attempts with the CVS failed to intubate. There was no significant difference in number of attempts: *p* value = 0.997 (chi-square test).

**Table 2:** **The total time of all attempts in seconds**

	**Median**	**Minimum**	**Maximum**	**95%** **confidence interval**
DL	15.00	4.00	104.50	15.99 to 23.00
CVS	14.50	6.10	61.00	14.53 to 17.99

There was no significant difference between techniques: *p* = 0.104 (Wilcoxon signed-rank test).

**Table 3:** **The first attempt times in seconds**

	**Median**	**Minimum**	**Maximum**	**25th percentile**	**75th percentile**	**90th percentile**
DL	15.00	4.00	53.17	11.47	20.40	33.80
CVS	14.50	6.10	40.00	12.18	18.10	22.08

There was no significant difference between techniques: *p* value = 0.425 (Mann-Whitney test).

**Table 4:** **Demographic factors** (***n*** = **81 participants**)

**Factors**	**Values**		
Sex	Male = 85.2% (69)	Female = 14.8% (12)	
Agency type	Fire = 17.3% (14)	Private = 63% (51)	
	Volume = 8.6% (7)	Municipal = 11.1% (9)	
Age	Median =34		Maximum = 65
Handedness	Right = 86.4% (70)	Left = 9.9% (8)	Ambi. = 3.7% (3)
Eye dominance	Right = 46.9% (38)	Left = 14.8% (12)	Unknown = 35.8% (29)

Survey results revealed CVS a median satisfaction rating of 9 (mean = 8.54 SD ± 1.827). The participants recorded a median CVS usefulness rating of 9 (mean = 8.47 SD ± 1.946). The tutorial received a median rating of 10 (mean = 9.12 SD ± 1.298) (Table 5).

**Table 5:** **Participant survey results** (**0** to **10 rating scale**)

	**Median**	**Minimum**	**Maximum**
CVS satisfaction	9	2	10
CVS usefulness	9	2	10
Tutorial quality	10	5	10

The median age for participants was 34 years old (range: 21 to 65 years old). The majority of participants were male. Agency type was predominantly private. Most (86.4%) were right handed, and eye dominancy was split by responses with 46.9% right, 14.8% left, and 35.8% unknown. There were no significant differences when analyzed by demographics (Table 6).

**Table 6:** **Significance of demographic factors relative to the total time of attempts**

**Factor**	**DL (** ***p*** **value)**	**CVS (** ***p*** **value)**	**Statistical test used**
Sex	0.878	0.175	Mann-Whitney test
Agency type	0.221	0.061	Kruskal-Wallis test
Handedness	0.287	0.058	Kruskal-Wallis test
Eye dominance	0.481	0.481	Kruskal-Wallis test

No demographic factors showed significance.

## Discussion

The results of the study show that the CVS was successfully utilized by almost every ALS EMTs who participated. The advantages of the design of a semi-rigid optical stylet should have a positive affect on intubation in floor-level positioning, and this may have some effect on the comparison to DL in this particular study. In a recent study, time to intubate patients in cardiac arrest was observed and time with DL was reported as a median of 15.8 s [11]. This result correlates well with the results of this study.

One provider was unable to successfully intubate with the CVS. This outlier is interesting because they were able to intubate with DL in 8 s on the first attempt. Not surprisingly, they rated their satisfaction with the device at 5, usefulness at 4, and the tutorial at 5. Alternatively, both participants that required three attempts to intubate with DL were able to successfully intubate with CVS and one of them did so with a time in the first quartile seemingly indicating ease of use of the CVS. That particular participant was also well over the median age of the group.

Although the CVS is in use with an agency in the study region, none of the ALS EMTs in the study had previous training or clinical experience with the CVS prior to the data collection. Based on the data, ALS EMT intubation performance was equal between the two devices. Although expected by the authors, this result is somewhat interesting in that DL is a skill that all participants practice and are required to show proficiency in on a regular basis. Their familiarity with this technique, and presumably the airway mannequin used in the study, could lead one to believe that DL should have been significantly faster overall. A number of factors may be at work to explain this finding; however, ease of use of the CVS and the capacity for ALS EMTs to improvise and adapt to new situations may account for these results. A study by Yun et al. showed that a group of paramedics were able to achieve similar times using DL as compared to an optical device and a video laryngoscope after only limited instruction [12]. In a previous study, the CVS in the hands of emergency physicians showed significantly positive results in a difficult airway model when compared to DL after limited instruction on the use of the device [13].

The results of this study in no way should be interpreted to imply CVS can be successfully deployed in the prehospital clinical environment without structured competency assessments that are customary to the introduction of new techniques and technology into patient care; however, the study suggests limited training is likely required. Times and the first pass success rate with the CVS may have been better if participants had prior experience with the device or a more in-depth training on its use.

Survey results were favorable toward the CVS with mean scores reflected satisfaction and perceived usefulness based on this brief exposure to the device. Some additional participant comments noted by the study authors during data collection included positive remarks about improved posture, ability to work in smaller spaces, and intubate patients on the ground without having to lay down, and the frequent comment that the ability to easily reenter the oropharynx to visualize the ETT in place with the device and show others (including receiving facility physicians) was a significant positive characteristic of the CVS.

### Current study limitations

Our sample of ALS EMTs was a convenience sample; however, we feel that the results would likely not differ if a random sampling had been used. Participants may have had too little time with the CVS to acquire a proper level of familiarity with the device in order to ensure a proper comparison to DL, a skill they regularly utilize and maintain through practice on a mannequin similar to the one used in this study. One of the presumed values of this type of device is the design advantage that should benefit ALS EMTs during a difficult airway scenario; however, this was not evaluated in this study. This device should be directly compared to DL and video laryngoscopy (VL) in future studies, as both DL and VL have been studied in the prehospital clinical environment. There may also be a potential difference in the outcomes between groups or ALS providers if years of experience had been evaluated. Potential associations with handedness and eye dominance may not be completely valid, as these values were self-reported and were not directly tested in this study.

## Conclusions

ALS EMTs with no previous practice experience with the CVS, and only a brief introductory tutorial, were able to successfully complete a simulated field intubation with a video-assisted semi-rigid fiberoptic stylet. There was no significant difference in the time required to successfully intubate when comparing DL and CVS techniques implying a similar level of competence when evaluated by this model. The survey results imply a willingness to consider integration of this type of device into their practice. Further study of this device in a simulated field environment with a high-fidelity simulator for evaluation of ALS EMT performance with the CVS in a difficult airway model is appropriate. Field implementation of the CVS, as with all critical skills, should include formal training and continued verified competency to ensure the best possible clinical utilization.

## Abbreviations

ALS: advanced life support
CVS: Clarus Video System
DL: direct laryngoscopy
ETT: endotracheal tube
EMT: emergency medical technician
VL: video laryngoscopy
